# Supplementary material for: Chromothripsis during telomere crisis is independent of NHEJ, and consistent with a replicative origin
Source: Genome Res. 2019 May;29(5):737–49. doi: 10.1101/gr.240705.118 (PMC6499312; doi:10.1101/gr.240705.118)
Supplement: Supplemental Material [file supp_gr.240705.118_Supplemental_file_1.zip › contigs/annotated_contigs/DB46/contig.2.DB46_length_667_mean_cov_5.39730134933.docx]

**DB46_length_667_mean_cov_5.39730134933**

```
TTATTTCTCATTTATGAGTCTGTGGTTGGCAGCACTTCTTCTTGGCTCTATTCTGTGCATCTCTTATTCCAAGACCTAGGCTGAAGCAG
 >chr3:59818130-59818413 - E=2e-158 p=4e-02
AGAAGCCCTCATCGGGGCCATGCTATTCACAATGCAGAGGACAAAAATGCAGAAAGTTAAGCCTTGCAACCTCATTTAAAGATTCTATG

TAGACATGGATATTTCTTATCTGCTTATAGTCCTTTGGGCAAAGAAAGTCACATAACCAAGCCAGACAATGTGGCTGGATGTTACAGAT

AAGGTAGGGGTGT|ATT|TTACAGATAGTAGAATTACAGCATGAAATATGTGTAACTTTCCCTTGGTTACCCAGTTAGTAAGTGTCAGA
              >chr3:59706986-59707373 - E=4e-218
GCTTTCATTTGAATCCAGATGATATAAATCCAGAGTCCACAATGGTACCTTTCTGGCTGAGATGAAAACCCAAGTCAACCAAACTCTAA

AGCCCCATCTCCCTGCTGCCCTGAACTTTCTCCTATGGAAAGAACCTAGTGGGCCTCTTTCTCTCATAAAGCTACTTAAAGACTGAAGA

CTAAAGCTAAAATGTTCCCTAGCACGCTCTTTATCCCTGTGGAGATCTTGTCTTTGTGCATTCATAGAGAATCTCACAAACTAATGCAT

CCAGACATCTGGCTTCAAGGGAAGGTAGATCTACTTTGGAAGTAAG
```
